# Anatomy of the infraorbital artery and its orbital branch

**DOI:** 10.1038/s41433-025-03671-y

**Published:** 2025-02-06

**Authors:** Jessica Y. Tong, Jeffrey Sung, WengOnn Chan, Alkis J. Psaltis, Dinesh Selva

**Affiliations:** 1 South Australian Institute of Ophthalmology, Royal Adelaide Hospital, Adelaide, SA, Australia; 2 Discipline of Ophthalmology and Vision Sciences, University of Adelaide, Adelaide, SA, Australia; 3 Department of Otolaryngology Head and Neck Surgery, Queen Elizabeth Hospital, Woodville, SA Australia; 4 Department of Surgery-Otolaryngology, Head and Neck Surgery University of Adelaide, Adelaide, SA Australia; 5 Faculty of Health and Medical Sciences, The University of Adelaide, Adelaide, SA Australia

**Keywords:** Anatomy, Health care

## Abstract

**Background/objectives:**

To characterise the infraorbital artery (IOA) and its orbital branch, which are key structures encountered during inferior orbital explorations, with potential for orbital haemorrhage and vision loss if inappropriately handled.

**Methods:**

Thirteen embalmed heads (26 orbits) were dissected. The following parameters were measured: orientation of the IOA in relation to the infraorbital nerve (ION); presence or absence of the orbital branch of the IOA; and the distance between the orbital branch of the IOA to the inferior orbital rim.

**Results:**

In the pterygopalatine fossa, the orientation of the IOA relative to V2 was medial (*n* = 9, 34.6%), inferior (*n* = 4, 15.4%), lateral (*n* = 4, 15.4%), inferolateral (*n* = 3, 11.5%), superolateral (*n* = 3, 11.5%), inferomedial (*n* = 2, 7.7%) and superior (*n* = 1, 3.8%). In the infraorbital canal, the IOA in relation to the ION was as follows: superomedial (*n* = 12, 46.2%), medial (*n* = 9, 34.6%), superior (*n* = 2, 7.7%), inferomedial (*n* = 2, 7.7%) and superolateral (*n* = 1, 3.8%). An orbital branch of the IOA was identified in 21/26 orbits (80.8%). The mean distance of the orbital branch to the inferior orbital rim was 13.0 ± 4.8 mm (range 2.0-23.0 mm).

**Conclusions:**

The IOA is an important vascular structure to recognise during inferior orbitotomies. The most common configuration is an IOA that runs medially to V2 in the pterygopalatine fossa, then superomedially to the ION within the infraorbital canal. The orbital branch of the IOA emerges 13 mm posterior to the inferior orbital rim. Recognition of these arterial branches and appropriate cauterization are paramount for avoiding significant operative complications.

## Introduction

The orbital floor is a surgical corridor with relevance across multiple surgical specialties. It provides a route of access for orbital floor fracture repair, orbital floor decompression, and exploration of the inferior orbit. It is therefore paramount that surgeons are knowledgeable of vascular structures and their variants within the inferior orbit. The infraorbital artery (IOA) travels within the inferior orbital fissure and infraorbital canal, and often emits an orbital branch along the latter segment. Inadvertent trauma could result in profuse bleeding, with risks of vision loss and impaired visualisation of the surgical field. The aim of this study was to describe the anatomy of the infraorbital artery and its orbital branch to inform inferior orbital surgical approaches.

## Materials and methods

**Fig. 1:**
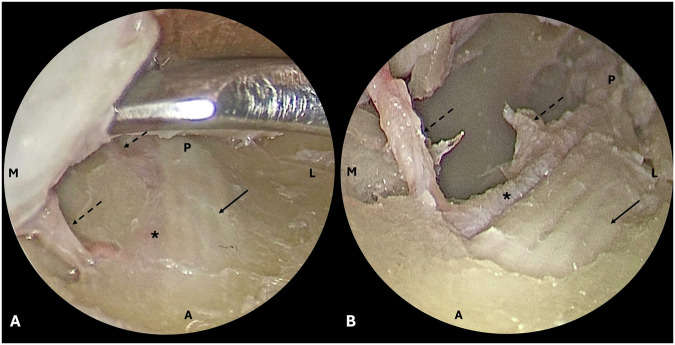
Cadaveric dissection demonstrating two orbital branches of the infraorbital artery. The infraorbital nerve (ION, solid arrow), infraorbital artery (IOA, asterisk) and orbital branches of the IOA (dashed arrows) are demonstrated. A anterior, L lateral, M medial, P posterior. **A** Orbital floor dissection in the subperiosteal plane demonstrates the ION within its canal, with the IOA running medially. **B** Once the roof of the infraorbital canal was removed, the IOA and its two orbital branches could be clearly identified travelling medial to the ION.

Ethics approval was obtained from the institutional review board prior to undertaking this study. This study adhered to the tenets of the Declaration of Helsinki. Thirteen formalin-fixed cadaver heads (26 orbits) were dissected. Red and blue coloured silicone was injected into the bilateral common carotid arteries and internal jugular veins respectively. A swinging eyelid approach was performed to reach the inferior orbit in the preseptal plane, followed by a subperiosteal dissection beyond the inferior orbital rim. The course of the IOA in relation to the infraorbital nerve (ION) within the infraorbital canal was recorded. The presence or absence of the orbital branch of the IOA was noted in each dissection. The distance between the orbital branch of the IOA and a point on the inferior orbital rim, above the infraorbital foramen, was measured. If there was more than 1 orbital branch of the IOA, the distance from the inferior orbital rim was taken from the more anterior branch. An example of the IOA and its orbital branch identified during dissection, is demonstrated in Fig. 1.

A two-tailed unpaired t-test was performed to compare the distance of the orbital branch from the inferior orbital rim between right and left sides, when present bilaterally. Statistical analysis was performed using GraphPad Prism Software. A *p*-value less than 0.05 was considered to be statistically significant.

## Results

The results of this study are summarised in Tables 1–3. In the pterygopalatine fossa, the IOA was noted to traverse in the following positions relative to the second branch of the trigeminal nerve (V2): medial (*n* = 9, 34.6%), inferior (*n* = 4, 15.4%), lateral (*n* = 4, 15.4%), inferolateral (*n* = 3, 11.5%), superolateral (*n* = 3, 11.5%), inferomedial (*n* = 2, 7.7%) and superior (*n* = 1, 3.8%). The orientation of the IOA in relation to the ION in the infraorbital canal was recorded as follows: superomedial (*n* = 12, 46.2%), medial (*n* = 9, 34.6%), superior (*n* = 2, 7.7%), inferomedial (*n* = 2, 7.7%) and superolateral (*n* = 1, 3.8%).

**Table 1:** The orientation of the infraorbital artery in relation to V2 was characterised, within the pterygopalatine fossa and the infraorbital canal.

Orientation of IOA relative to V2	Number	Percentage
**Pterygopalatine fossa**		
Superior	1	3.8%
Superolateral	3	11.5%
Medial	9	34.6%
Inferior	4	15.4%
Inferomedial	2	7.7%
Inferolateral	3	11.5%
Lateral	4	15.4%
**Infraorbital canal**		
Superior	2	7.7%
Superomedial	12	46.2%
Superolateral	1	3.8%
Medial	9	34.6%
Inferomedial	2	7.7%

**Table 2:** Dimensions of the orbital branch of the infraorbital artery were measured.

Orbital branch	Number	Percentage
Present	21	80.8%
Absent	5	19.2%
	**Mean** **±** **SD (mm)**	**Range**
**Distance to inferior orbital rim**	13.0 ± 4.8	2.0-23.0

**Table 3:** In the 10 specimens with bilateral orbital branches, there was no significant difference between sides, in the distance from which the orbital branch arose to the inferior orbital rim (*p* = 0.26).

Right side (mm)	Left side (mm)
11.0	11.5
14.0	14.5
2.0	12.5
10.0	15.0
14.0	13.0
7.0	6.5
18.0	16.5
20.0	23.0
10.0	12.0
10.0	16.0

The orbital branch of the IOA was identified in 21/26 orbits (80.8%). It was absent in both orbits in 2 specimens and unilaterally absent in 1 specimen. Its mean distance from the inferior orbital rim was 13.0 ± 4.8 mm (range 2.0–23.0 mm). There were 3 orbits that had two orbital branches of the IOA; this occurred bilaterally in 1 specimen and unilaterally in 1 specimen. In the bilateral case, the more anterior branch was located 7 mm and 6.5 mm, while the more posterior branch was 12.5 mm and 13.5 mm, posterior to the orbital rim. In the unilateral case, the orbital branches were encountered 11.5 mm and 16 mm from the orbital rim. In the 10 specimens (20 orbits) with orbital branches present bilaterally, a comparison of its distance from the inferior orbital rim between right and left sides demonstrated that there was no significant difference (*p* = 0.26).

## Discussion

This cadaveric dissection study represents one of the largest to date to investigate the IOA and its orbital branch. The indications for an inferior orbital exploration are broad and encompass fracture repair, orbital floor reconstruction, decompressions and inferior orbital pathologies that may necessitate biopsy, debulking or en bloc excision. From an endonasal perspective, any associated vascular bundle is important to identify during approaches to the ION and maxillary roof. The IOA and its orbital branch are therefore key structures to recognise and avoid during any of these surgical explorations. The aim of this study was twofold: to determine the position of the IOA in relationship to the ION, and secondly to determine the presence and position of its orbital branch.

The IOA branches from the internal maxillary artery in the pterygopalatine fossa, where it ascends to reach the posteromedial aspect of the inferior orbital fissure, before emerging with the ION within the infraorbital canal [1]. The relationship between the IOA and ION has been investigated in several cadaveric dissection studies. At the transitional point between the pterygopalatine and orbitomaxillary segments of the ION, the IOA was often situated inferior or posterolateral to the nerve (75%), although other variants included an anteromedial position (15%) and a high loop anterior to the nerve (10%) [2]. In contrast, in this study, there were many variations of the IOA’s position relative to V2. The most common configuration was the IOA travelling medially to V2 in the pterygopalatine fossa upon branching from the internal maxillary artery, in 34.6% of cases. Li et al. reported that the IOA continued medial to the ION in 75% of cases when viewed within the roof of the maxillary sinus, from an endonasal prelacrimal approach [3]. Other variations of the IOA in relation to the ION were superior, inferior or intraneural positions where the artery traversed between ION fibres [3]. Kazkayasi et al. reported similar data in their cadaveric work, where the IOA was consistently found to be superomedial to the ION upon its exit at the infraorbital foramen [4]. This was consistent with our results, where the IOA was most commonly superomedial to the ION in the infraorbital canal.

The orbital branch of the IOA is an important orbital floor landmark and has been described in select orbital and ophthalmic anatomical texts [1, 5–7]. In this study, the orbital branch was present in 21 out of 26 orbits (80.8%), which reflects the experiences described in Patel et al.’s cadaveric study which demonstrated its presence in 8 of 9 orbits [8]. However, in a clinical study of orbital floor explorations, the orbital branch was present in 10 consecutive cases [9]. These disparate results likely represent sampling bias, though indicate that the orbital branch is found in the majority of cases. Histopathological studies have confirmed that the orbital branch traverses within a neurovascular bundle, accompanied by a small vein and peripheral nerve [8].

Much of the work regarding the orbital branch of the IOA has stemmed from experiences with significant orbital haemorrhage that were ultimately deemed preventable [9–11]. In these papers, the indications for accessing the orbital floor were for reconstructive purposes following a floor blowout fracture, medial wall fracture accessed via a subciliary approach, and maxillary atelectasis. The timing of haemorrhage was variable, occurring either immediately upon inadvertent intraoperative trauma, or 10 minutes to 15 hours postoperatively [9–11]. This demonstrates the importance of anticipation and planned cauterisation. Notably, the orbital branch of the IOA supplies multiple structures, including the inferior oblique, inferior rectus, orbital floor contents including orbital fat, and lacrimal sac from terminal branches [1, 6, 8, 12]. However, dividing the orbital branch is unlikely to compromise vascular supply of the relevant extraocular muscles and orbital fat, due to the abundance of internal and external carotid anastomoses with the inferior muscular branch of the ophthalmic artery [1]. Division of the orbital branch can be performed with bipolar electrocautery, which is preferable to monopolar cautery to minimise thermal damage to the ION [9].

In this study, the orbital branch of the IOA emerged at an average distance of 13.0 ± 4.8 mm (range 2.0-23.0 mm) posterior to the inferior orbital rim, which is consistent with data derived from prior cadaveric work. In two separate cadaveric dissections, the orbital branch emerged at a mean distance of 16.6 mm (range 10.0-23.0 mm) and 14.1 mm (range 3.0-20.0 mm) from the orbital rim [8, 13]. Similarly, clinical studies of inferior orbitotomies have reported that the orbital branch appears along the infraorbital canal at a mean distance of 14.4 mm (range 13.0-17.0 mm) posterior to the rim [9, 10]. In this study, there was no significant difference in the distance from the orbital branch to the inferior orbital rim between sides, when bilateral orbital branches were present. This would indicate some degree of anatomical symmetry in the arterial branching pattern. However, while most specimens were consistent with this observation, there was one cadaver with orbital branches that arose 2.0 mm and 12.5 mm from the inferior orbital rim (Table 3). The power of the statistical analysis would be improved with a larger sample size.

While the orbital branch usually emerges as a solitary vessel, there may also be multiple branches. We had 3 instances of two orbital branches arising from the IOA, with one specimen demonstrating this variant bilaterally. Similarly, Chien et al.’s cadaveric study found that 5 of 14 orbits had 1-2 additional branches of the IOA accompanying smaller branching nerves of the ION [13]. These measurements are supported by intraoperative clinical observations, where multiple branches may appear as 2-3 vessels appearing in 3-4 mm increments along the infraorbital canal [10]. Finally, the orbital branch can be variable in its configuration, and may emerge from the IOA as a thick band or a thin duplicate structure [8]. Either way, it can masquerade as a band of connective tissue and upwards retraction of the orbital contents should clearly present the arterial vessel for cauterisation.

We acknowledge that there are limitations inherent to a cadaveric dissection study. The IOA and its orbital branch were readily identified as being distinct from the adjacent ION, but red silicone was nevertheless injected to confirm the arterial structures. In 5 out of 26 orbits, no orbital branch was identified, which was a higher proportion than data reported from other cadaveric and clinical studies. This may have been a true reflection of significant anatomical variations. However, the integrity of a fragile orbital branch may have become compromised during the silicone injection or fixation process. In addition, further clinical data from inferior orbitotomies is necessary to validate our cadaveric measurements.

In conclusion, the IOA and its orbital branch are important arterial structures that should be properly anticipated and managed during any inferior orbitotomy. Cadaveric dissections in this study demonstrated that the IOA commonly traversed medially to V2 in the pterygopalatine fossa, then moved superomedially to the ION within the infraorbital canal. The orbital branch of the IOA emerged on average 13 mm from the inferior orbital rim, but multiple arterial branches was also occasionally observed. Careful dissection and handling of these structures is key to prevention of orbital haemorrhage and preservation of the surgical field.

## Summary

### What was known before

- There is heterogeneous data on the position of the infraorbital artery in relation to V2 within the pterygopalatine fossa and orbital floor.
- The orbital branch of the infraorbital artery is an important landmark in orbital floor explorations. There is heterogeneous data on its prevalence.

### What this study adds

- This is the one of the largest cadaveric studies to date to characterise the infraorbital artery’s orientation in relation to V2, and its arterial branching pattern.
- The infraorbital artery is most commonly medial to V2 in the pterygopalatine fossa, followed by a superomedial position to the infraorbital nerve within the canal.
- The orbital branch was present in 80.8% of orbits dissected, and its distance to the inferior orbital rim was 13.0 mm.

## Supplementary information

Eye Reporting Checklist
